# Predictive value of qSOFA score for death in emergency department resuscitation room among adult trauma patients:a retrospective study

**DOI:** 10.1186/s12873-021-00498-0

**Published:** 2021-09-10

**Authors:** Wenjuan Huang, Peng Yang, Feng Xu, Du Chen

**Affiliations:** 1 Department of Critical Care Medicine, the First Affiliated Hospital of Soochow University, Suzhou City, Jiangsu Province, China; 2 Department of Emergency Medicine, the First Affiliated Hospital of Soochow University, Suzhou City, Jiangsu Province, China

**Keywords:** qSOFA, Predictive value, ED resuscitation room, Trauma patients

## Abstract

**Background:**

To explore the predictive value of the quick Sequential Organ Failure Assessment (qSOFA) score for death in the emergency department (ED) resuscitation room among adult trauma patients.

**Methods:**

During the period November 1, 2016 to November 30, 2019, data was retrospectively collected of adult trauma patients triaged to the ED resuscitation room in the First Affiliated Hospital of Soochow University. Death occurring in the ED resuscitation room was the study endpoint. Univariate and multivariate analyses were performed to explore the association between qSOFA score and death. Receiver operating characteristic (ROC) curve analysis was also performed for death.

**Results:**

A total of 1739 trauma victims were admitted, including 1695 survivors and 44 non-survivors. The death proportion raised with qSOFA score: 0.60% for qSOFA = 0, 3.28% for qSOFA = 1, 12.06% for qSOFA = 2, and 15.38% for qSOFA = 3, *p* < 0.001. Subgroup of qSOFA = 0 was used as a reference. In univariate analysis, crude OR for death with qSOFA = 1 was 5.65 [95% CI 2.25 to 14.24, *p* < 0.001], qSOFA = 2 was 22.85 [95% CI 8.84 to 59.04, *p* < 0.001], and qSOFA = 3 was 30.30 [95% CI 5.50 to 167.05, p < 0.001]. In multivariate analysis, with an adjusted OR (aOR) of 2.87 (95% CI 0.84 to 9.87, *p* = 0.094) for qSOFA = 1, aOR 6.80 (95% CI 1.79 to 25.90, *p* = 0.005) for qSOFA = 2, and aOR 24.42 (95% CI 3.67 to 162.27, *p* = 0.001) for qSOFA = 3. The Area Under the Curve (AUC) for predicting death in the ED resuscitation room among trauma patients was 0.78 [95% CI, 0.72–0.85].

**Conclusions:**

The qSOFA score can assess the severity of emergency trauma patients and has good predictive value for death in the ED resuscitation room.

**Supplementary Information:**

The online version contains supplementary material available at 10.1186/s12873-021-00498-0.

## Background

Trauma continues to be an important public health problem worldwide, including in China. With the increase of the Chinese economy, severe and mass traumas caused by high-energy factors such as traffic accidents and falling from a height have an increasing incidence. Officially reported rate of road traffic injuries, disability and deaths increased significantly with rapid motorization beginning in the late 1980s, with road traffic injuries and corresponding deaths taking a significant threat on China’s population health [1].

The World Health Organization data showed that the incidence of road traffic fatality (18.8/100000 population) in China was higher than the average for developed and developing countries (9.2 and 18.4 deaths per 100,000, respectively) [2]. Globally, road traffic accidents are the leading causes of death among young people, particularly among young people aged 15–29 years [2].

An appropriate score to predict the mortality risk in trauma cases is necessary. In the last 40 years, a variety of Anatomical Scoring Systems, Physiological Scoring Systems, Combination of Anatomic and Physiological Scoring Systems (mixed scores), represented respectively by Injury Severity Score (ISS), Revised Trauma Score (RTS), Trauma and Injury Severity Scores (TRISS), had been developed to indicate severity of trauma, assess the prognosis and guide the therapeutic strategy among trauma victims [3–5].

The variables that are taken into consideration in the RTS can all be evaluated at the bedside, but calculation relies on formulas that are too complicated to be used in the ED resuscitation room. While anatomical scores and mixed scores describe all the injuries recorded by clinical examination, imaging, surgery or autopsy, because of complicated calculation, the ISS, RTS and TRISS may not be appropriate for use in the ED resuscitation room.

Experts and scholars are still innovating to develop other relatively simple scores, such as Mechanism, Glasgow Coma Scale, Age, and Arterial Pressure (MGAP), Glasgow Coma Scale, Age, and Systolic Blood Pressure score (GAP), New Trauma Score (NTS) and Trauma Rating Index in Age, Glasgow Coma Scale, Respiratory rate and Systolic blood pressure score (TRIAGES) [6–10]. However, few studies have demonstrated the validity of these trauma scores in the ED resuscitation room in China. A convenient tool for initial severity estimation of trauma that does not require sophisticated medical tests or devices and is especially useful in ED resuscitation room is wanted.

The qSOFA score has been recommended as a simple and quick tool to estimate risk of complications in patients outside the Intensive Care Unit (ICU) with suspected infection [11–13]. The qSOFA score has also been utilized to predict mortality risk in patients without suspected infection [14–18].

The intention of this study was to investigate the correlation between qSOFA score and death in the ED resuscitation room among trauma patients. It was hypothesized that the qSOFA score would be associated with injury severity among trauma patients and could be used as a good predictor for mortality.

## Methods

### Study design, setting

This retrospective study was performed in the First Affiliated Hospital of Soochow University, Suzhou, China. Death occurring in the ED resuscitation room was the study endpoint. In the present study, trauma patients were divided into 2 groups as survivor and non-survivor, then divided into 4 subgroups according to qSOFA score at presentation to the ED resuscitation room. The validity of qSOFA score for predicting death in the ED resuscitation room among trauma cases was explored.

### Selection of participants and data collection

Adult patients who underwent traumas and were triaged to the ED resuscitation room between November 1, 2016 and November 30, 2019 were included. Trauma patients triaged to consulting rooms and/or without complete information that the study required were excluded.

From the emergency clinical information system (ECIS) [Meehealth Information Technology Co., Ltd. (Shanghai, China)], the following data was collected: demographics (age, sex), initial vital signs (systolic blood pressure (SBP), respiratory rate (RR), pulse rate, temperature, and oximetry), level of consciousness and Glasgow Coma Scale scores (GCS) at presentation, death time in ED resuscitation room.

The qSOFA score (range, 0–3 points) consists of three elements, assigning one point each to: SBP of 100 mmHg or less, RR of 22/min or greater, and altered mentation [11, 13]. The RTS as a tool for evaluation of trauma outcome is calculated by adding the coded values of GCS, SBP and RR [4].

### Statistical analysis

Continuous variables were tested for normality using the Shapiro–Wilk test. Continuous variables failing to conform to normality were expressed as median (IQR) and compared using the Mann-Whitney test. Categorical variables were expressed as frequencies and percentages and compared using the Likelihood-ratio Chi squared test. Spearman correlation was used to evaluate relationships of variables. Logistic regression models were performed to calculate the odds ratios (ORs) of variables for death. A ROC curve was performed to evaluate the AUC of predictor for death. Statistical analyses and graphics were completed with STATA (StataCorp.2017. Stata Statistical Software: Release 15. College Station, TX: Stata Corp LLC). Two-tailed *P* < 0.05 was considered to be statistically significant.

## Results

Demographic and clinical data are shown in Table 1. During the study period, there were 1739 admissions. There were no significant differences in sex and age between the survivor and non-survivor group (*P* > 0.05). Significant difference was found in the length of stay in the ED resuscitation room between groups (*P* < 0.001). In correlation analysis, qSOFA score was negatively associated with RTS (r = − 0.38, *p*<0.001).

**Table 1:** Baseline characteristics

Variables	Survivor 1695(97.47%)	Non-survivor 44(2.53%)	***P*** value
**Sex**			0.532
Female	457(26.96%)	10(22.73%)	
Male	1238(73.04%)	34(77.27%)	
**Age** (years)	51(25)	50(20)	0.757
**RTS**	12(0)	8(6)	< 0.001
**qSOFA**	0(1)	1(1)	< 0.001
**Hours in the ED**	4(13)	13(30)	< 0.001

Continuous variables were expressed as median (IQR); categorical variables were expressed as n/percentage; *P* values were calculated by Mann-Whitney test

**Fig. 1:**
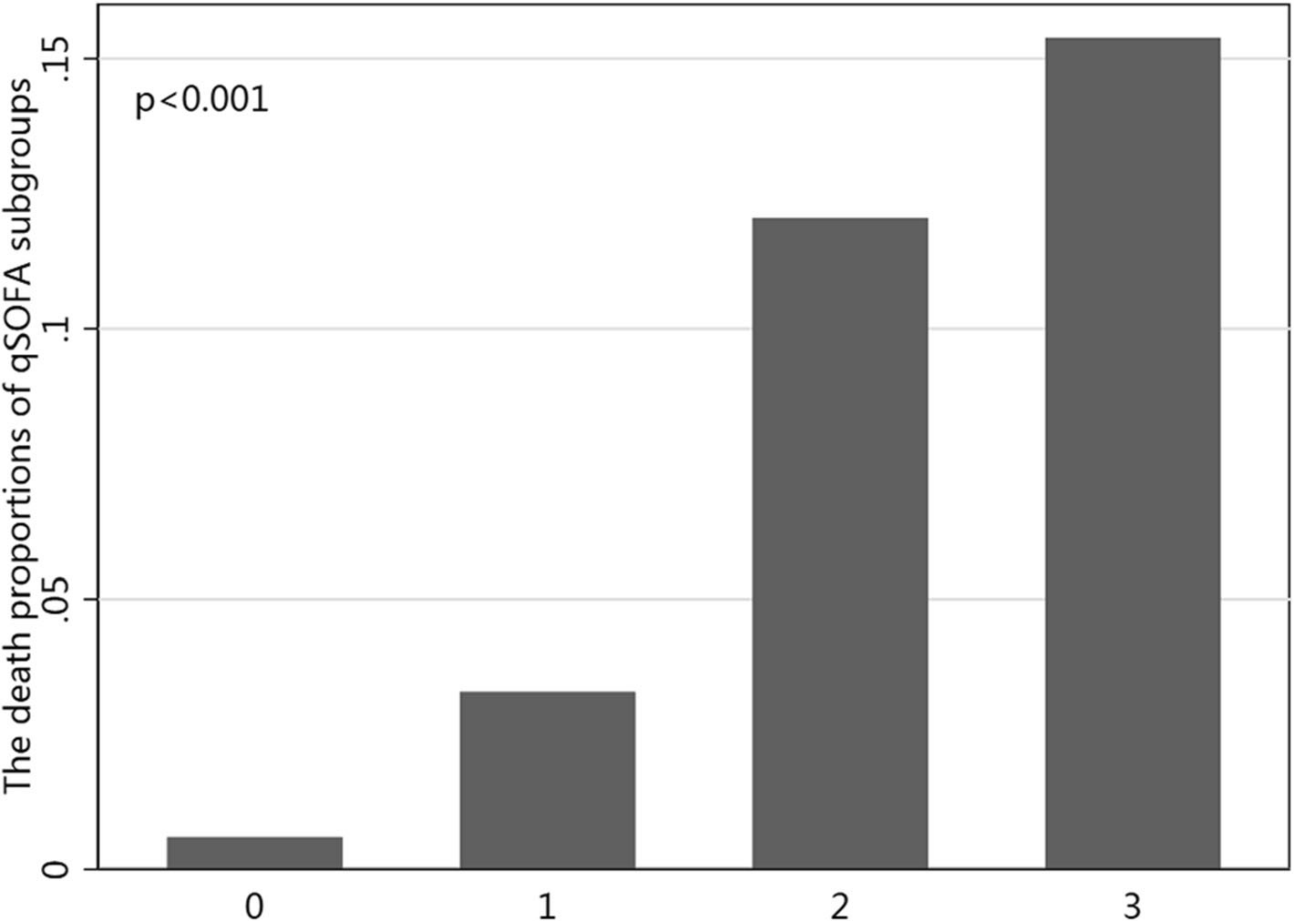
Barchart for the death proportions of qSOFA subgroups. The death proportions of the four qSOFA subgroups were significantly different (0.60, 3.28, 12.06 and 15.38%, *p* < 0.001)

Patients were divided into four subgroups according to qSOFA scores at presentation to the ED resuscitation room. Of the 1739 subjects, 1006 (57.85%) had a qSOFA score of 0, 579 (33.29%) had a score of 1, 141 (8.11%) had a score of 2, and 13 (0.75%) had a score of 3. The mortality rates according to qSOFA scores were compared by Likelihood-ratio Chi squared test. An analysis of qSOFA score associated with death proportion is illustrated in Fig. 1. The death proportion raised with qSOFA score: 0.60% (6/1006) for qSOFA = 0, 3.28% (19/579) for qSOFA = 1, 12.06%(17/141) for qSOFA = 2, and 15.38%(2/13) for qSOFA = 3, *p* < 0.001.

**Fig. 2:**
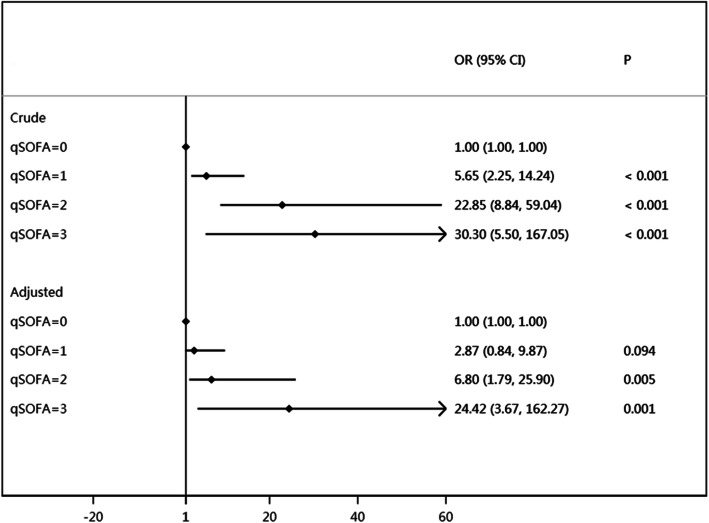
Forestplot of crude ORs and adjusted ORs of qSOFA. OR, odds ratio; RTS, Revised Trauma Score; crude ORs were calculated by univariable logistic regression model; aORs were calculated by multivariable logistic regression of qSOFA, sex, age and RTS

Subgroup of qSOFA = 0 was used as a reference. In univariate analysis (Fig. 2), crude OR for death with qSOFA = 1 was 5.65 [95% CI 2.25 to 14.24, *p* < 0.001], qSOFA = 2 was 22.85 [95% CI 8.84 to 59.04, *p* < 0.001], and qSOFA = 3 was 30.30 [95% CI 5.50 to 167.05, *p* < 0.001]. Given the association between elevated qSOFA scores and increased likelihood of death on univariate analyses, it was hypothesized whether this association would also present on multivariate analyses. In multivariate analysis (Fig. 2), the qSOFA score ≥ 2 was significantly associated with death, with an Adjusted OR (aOR) of 2.87 (95% CI 0.84 to 9.87, *p* = 0.094) for qSOFA = 1, aOR 6.80 (95% CI 1.79 to 25.90, *p* = 0.005) for qSOFA = 2, and aOR 24.42 (95% CI 3.67 to 162.27, *p* = 0.001) for qSOFA = 3. qSOFA scores of 2 or more were significantly associated with death after adjustment for other factors (gender, age, RTS).

**Fig. 3:**
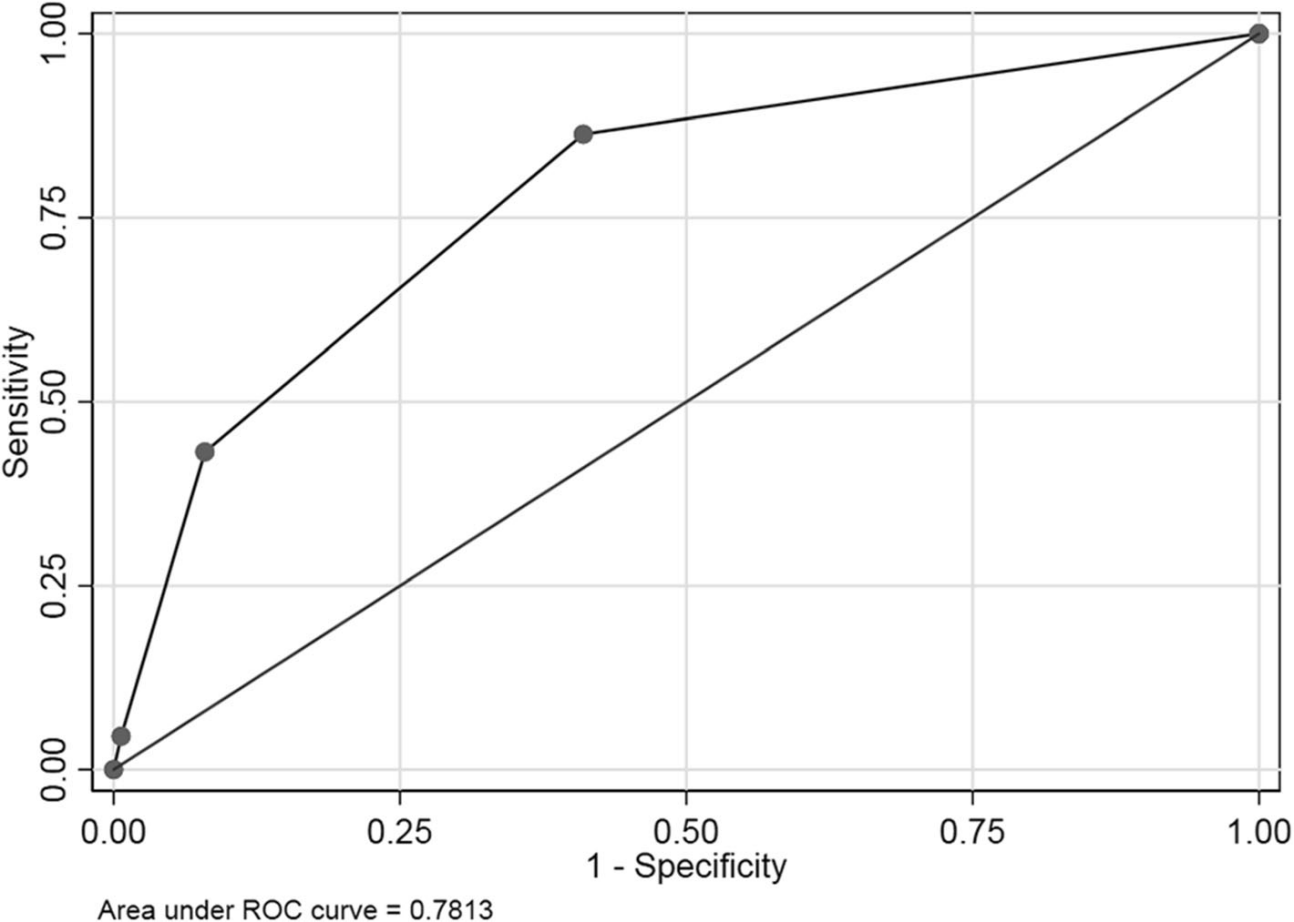
Receiver operating characteristic curve of qSOFA predicting death

A ROC curve was used to determine predictive capacity of qSOFA score for death (Fig. 3). The AUC for predicting death in the ED resuscitation room among trauma patients was 0.78 [95% CI, 0.72–0.85] (moderate predictive ability).

## Discussion

Predicting risk of death in the ED resuscitation room among trauma patients is the focus of clinical practice. A prediction tool that is accurate and easy-to-use is expected by clinicians.

The qSOFA, as a simple novel prediction score, is used to predict in-hospital mortality in non-ICU patients with suspected infection [11]. Studies have also verified the validity of qSOFA in predicting outcome of ED patients with and without suspected infections [18], as well as patients with burn, cancer, pesticide poisoning, and blunt trauma [14–17].

In the current study, a single center, retrospective study of adult trauma patients triaged to the ED resuscitation room was performed, with the purpose to explore the predictive validity of qSOFA for death in the ED resuscitation room in this cohort. This study demonstrates that qSOFA score has good correlation with death, with higher qSOFA score reflecting higher risk of death.

The RTS has been developed for use in triage and outcome prediction among injury patients [4]. High score of RTS indicates high survival probability [4]; but high score of qSOFA means low survival probability. The qSOFA score was negatively correlated with RTS (r = − 0.38, *p*<0.001). This result was consistent with that of Jawa et al. [17]. These data further suggest that high qSOFA score is indicative of a risk of an unfavorable prognosis following trauma.

In the present study, patients with a qSOFA score of 0 had a 0.6% incidence rate of death. As the qSOFA score increased from 1 to 3, the rate of death significantly increased from 3.28 to 15.38%. The death proportions were significantly higher in patients with qSOFA of 2 or more. It was found that elevated qSOFA scores were directly associated with increased death proportion in Likelihood-ratio Chi squared test. This tendency was in concordance with those reported by Singer et al. [18] and Jawa et al. [17]. In the research by Singer AJ et al., qSOFA scores were associated with mortality (0 [0.6%], 1 [2.8%], 2 [12.8%], and 3[25.0%]), whilst in the research by Jawa et al., qSOFA scores were associated with in-hospital mortality (1.7% with qSOFA equals to 0; 8.7% with qSOFA equals to 1; 22.4% with qSOFA equals to 2; 23.1% with qSOFA equals to 3; *p* < 0.001). SilvioA et al. [15] revealed that for patients with qSOFA score<2 vs qSOFA score ≥ 2, the hospital mortality rate was 7.36% vs 35.7% (28.3%; 95% CI, 13–47.7%, *p* < 0.001). In contrast, an interesting result was reported by A. Prasad, et al. [14], the highest mortality rate was in the group of qSOFA score = 2 (12.2%), with none in the group of score = 3, which is inconsistent with the present study finding, but this result was not discussed by authors.

As higher qSOFA score was more correlated with death on univariate analysis, its performance was examined by multivariate analysis. Using the multivariate logistic regression (after adjustment for sex, age, RTS), it was found that qSOFA scores of 2 and 3 were independently associated with death. It was cautiously suggested that clinicians should pay more attention and give more frequent monitoring to trauma patients with qSOFA scores of 2 or more at presentation.

Among patients with suspected infection in non-ICU settings, the AUC of qSOFA for predicting in-hospital mortality was 0.81 (95% CI, 0.80–0.82) [13]. Whilst in the present study, the AUC of qSOFA for predicting death was 0.78 [95% CI, 0.72–0.85]. The two predictive values are close. The performance of the qSOFA in the current study was also similar to that reported by Singer et al. [18], in which the AUC for predicting mortality among patients with and without suspected infection were 0.75 (95% CI 0.71 to 0.78) and 0.70 (95% CI 0.65 to 0.74), respectively.

In addition, this study’s objectives were confined to trauma patients triaged to the ED resuscitation room. Trauma victims triaged to consulting rooms were excluded, they were usually less severely ill and generally at lower risk of death. Further, the present study endpoint was death occurring in the ED resuscitation room. Death after leaving the ED resuscitation room was not covered by this study. These might lead to an underestimation of total death toll, and an over- or underestimation of death proportion, even an over- or underestimation of predictive capacity of qSOFA for death.

Several limitations exist in this present study. This was a retrospective study from a single center, which is subject to selection bias. The results may not be representative. In the future, large, multi-center retrospective reviews as well as prospective research may be required to determine whether the qSOFA scores can accurately predict death in the ED resuscitation room among trauma patients.

## Conclusions

The qSOFA score can assess the severity of emergency trauma patients and has good predictive value for death in the ED resuscitation room.

## Supplementary Information

**Additional file 1.**

## Data Availability

All data generated or analysed during this study are included in this article and its supplementary information files.

## Abbreviations

qSOFA: quick Sequential Organ Failure Assessment
ED: emergency department
ROC: Receiver operating characteristic
aOR: adjusted OR
AUC: Area Under the Curve
ISS: Injury Severity Score
RTS: Revised Trauma Score
TRISS: Trauma and Injury Severity Scores
MGAP: Mechanism, Glasgow Coma Scale, Age, and Arterial Pressure
GAP: Glasgow Coma Scale, Age, and Systolic Blood Pressure score
NTS: New Trauma Score
TRIAGES: Trauma Rating Index in Age, Glasgow Coma Scale, Respiratory rate and Systolic blood pressure score
ICU: Intensive Care Unit
SBP: systolic blood pressure
RR: respiratory rate
ECIS: emergency clinical information system
GCS: Glasgow Coma Scale scores
ORs: odds ratios

## References

[1] Jiang BG, Liang S, Peng ZR, Cong HZ, Levy M, Cheng Q, Wang T, Remais JV (2017). Transport and public health in China: the road to a healthy future. Lancet.

[2] World Health Organization. Global status report on road safety 2015. Geneva, Switzerland 2015, page x, 5, 110. ISBN 978 92 4 156506 6.

[3] Baker SP, Oʼneill B, Haddon W, Long WB (1974). The injury severity score: a method for describing patients with multiple injuries and evaluating emergency care. The Journal of Trauma: Injury, Infection, and Critical Care.

[4] Champion HR, Sacco WJ, Copes WS, Gann DS, Gennarelli TA, Flanagan ME (1989). A revision of the trauma score. The Journal of Trauma: Injury, Infection, and Critical Care..

[5] Boyd CR, Tolson MA, Copes WS (1987). Evaluating trauma care: the TRISS method. J Trauma.

[6] Raum MR, Nijsten MWN, Vogelzang M, Schuring F, Lefering R, Bouillon B, Rixen D, Neugebauer EA, ten Duis H, Polytrauma Study Group of the German Trauma Society (2009). Emergency trauma score: an instrument for early estimation of trauma severity*. Crit Care Med.

[7] Sartorius D, Le Manach Y, David J, Rancurel E, Smail N, Thicoïpé M (2010). Mechanism, Glasgow coma scale, age, and arterial pressure (MGAP): a new simple prehospital triage score to predict mortality in trauma patients*. Crit Care Med.

[8] Kondo Y, Abe T, Kohshi K, Tokuda Y, Cook EF, Kukita I (2011). Revised trauma scoring system to predict in-hospital mortality in the emergency department: Glasgow coma scale, age, and systolic blood pressure score. Crit Care.

[9] Jeong JH, Park YJ, Kim DH, Kim TY, Kang C, Lee SH, Lee SB, Kim SC, Lim D (2017). The new trauma score (NTS): a modification of the revised trauma score for better trauma mortality prediction. BMC Surg.

[10] Shiraishi A, Otomo Y, Yoshikawa S, Morishita K, Roberts I, Matsui H (2019). Derivation and validation of an easy-to-compute trauma score that improves prognostication of mortality or the trauma rating index in age, Glasgow coma scale, respiratory rate and systolic blood pressure (TRIAGES) score. Crit Care.

[11] Singer M, Deutschman CS, Seymour CW, Shankar-Hari M, Annane D, Bauer M, Bellomo R, Bernard GR, Chiche JD, Coopersmith CM, Hotchkiss RS, Levy MM, Marshall JC, Martin GS, Opal SM, Rubenfeld GD, van der Poll T, Vincent JL, Angus DC (2016). The third international consensus definitions for sepsis and septic shock (Sepsis-3). J Am Med Assoc.

[12] Freund Y, Lemachatti N, Krastinova E, Van Laer M, Claessens Y, Avondo A (2017). Prognostic accuracy of sepsis-3 criteria for in-hospital mortality among patients with suspected infection presenting to the emergency department. J Am Med Assoc.

[13] Seymour CW, Liu VX, Iwashyna TJ, Brunkhorst FM, Rea TD, Scherag A, Rubenfeld G, Kahn JM, Shankar-Hari M, Singer M, Deutschman CS, Escobar GJ, Angus DC (2016). Assessment of clinical criteria for sepsis. J Am Med Assoc.

[14] Prasad A, Thode HC, Singer AJ (2020). Predictive value of quick SOFA and revised Baux scores in burn patients. Burns..

[15] Ñamendys-Silva SA, Joachin-Sánchez E, Joffre-Torres A, Córdova-Sánchez BM, Ferrer-Burgos G, González-Chon O, Herrera-Gomez A Usefulness of qSOFA and ECOG scores for predicting hospital mortality in postsurgical cancer patients without infection. International journal of chronic diseases. Volume 2019, Article ID 9418971, Usefulness of qSOFA and ECOG Scores for Predicting Hospital Mortality in Postsurgical Cancer Patients without Infection, 2019, 9418975, DOI: 10.1155/2019/9418971. 10.1155/2019/9418971 PMC6521516 31187034

[16] Cho YS, Chun BJ, Moon JM. The qSOFA score: A simple and accurate predictor of outcome in patients with glyphosate herbicide poisoning. Basic & clinical pharmacology & toxicology.2018;123:615–621. 10.1111/bcpt.13044 29786949

[17] Jawa RS, Vosswinkel JA, Mccormack JE, Huang EC, Thode HC, Shapiro MJ (2017). Risk assessment of the blunt trauma victim: the role of the quick sequential organ failure assessment score (qSOFA). Am J Surg.

[18] Singer AJ, Ng J, Thode HC, Spiegel R, Weingart S (2017). Quick SOFA scores predict mortality in adult emergency department patients with and without suspected infection. Ann Emerg Med.

